# CircSERPINA3 regulates SERPINA3-mediated apoptosis, autophagy and aerobic glycolysis of prostate cancer cells by competitively binding to MiR-653-5p and recruiting BUD13

**DOI:** 10.1186/s12967-021-03063-2

**Published:** 2021-12-03

**Authors:** Zengshu Xing, Sailian Li, Zhenxiang Liu, Chong Zhang, Zhiming Bai

**Affiliations:** 1 Department of Urology, Affiliated Haikou Hospital of Xiangya Medical College, Central South University, No. 43 Renmin Road, Meilan District, Haikou, 570208 Hainan China; 2 Department of Gastroenterology, Affiliated Haikou Hospital of Xiangya Medical College, Central South University, No.43 Renmin Road, Meilan District, Haikou, 570208 Hainan China

**Keywords:** circSERPINA3, SERPINA3, miR-653-5p, BUD13, Prostate cancer

## Abstract

**Background:**

Prostate cancer (PCa) belongs to an epithelial malignancy that occurs in the prostate gland and is the most common malignancy of the male genitourinary system. Referring to related literature, circSERPINA3 has been reported to be up-regulated in PCa. However, its biological function remains unclear.

**Purpose:**

This study aimed to reveal the specific role and relevant molecular mechanism of circSERPINA3 in PCa.

**Methods:**

RT-qPCR was used to examine gene expression and functional analyses were conducted to verify the effect of circSERPINA3 on cell apoptosis, autophagy and aerobic glycolysis in PCa cells. Mechanism assays were applied to evaluate the relationship among circSERPINA3/miR-653-5p/SERPINA3/BUD13.

**Results:**

CircSERPINA3 was verified to be up-regulated in PCa cells and to inhibit cell apoptosis while promoting aerobic glycolysis and autophagy in PCa cells. CircSERPINA3 and SERPINA3 were also testified to bind to miR-653-5p through a line of mechanism experiments. Moreover, it was discovered that circSERPINA3 could stabilize SERPINA3 mRNA via recruiting BUD13. Additionally, SERPINA3 was verified to inhibit cell apoptosis, while promoting aerobic glycolysis and autophagy in PCa cells.

**Conclusions:**

Our study suggested that circSERPINA3 regulated apoptosis, autophagy and aerobic glycolysis of PCa cells by competitively binding to miR-653-5p and recruiting BUD13.

**Graphic abstract:**

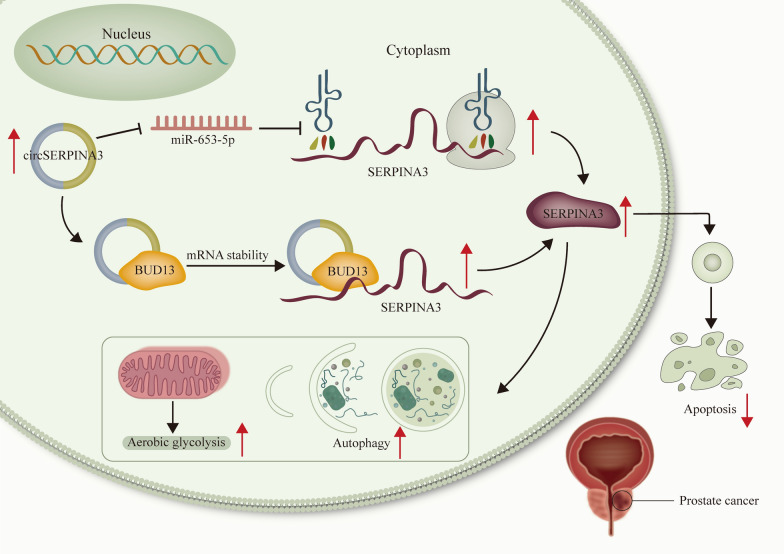

**Supplementary Information:**

The online version contains supplementary material available at 10.1186/s12967-021-03063-2.

## Background

Prostate cancer (PCa) is a heterogeneous disease with a variable natural history while optimal management of this disease remains demanding [1]. In China, research on PCa has been on an increasing trend, and personalized therapies based on genetic factors, nano-medicine and traditional Chinese medicine have been the focus of current PCa research [2]. Though novel biomarkers for PCa that can contribute to precision medicine in the near future have been identified in recent years [3], the treatment work for PCa is still hard to push, which is partly due to its delayed diagnosis.

Circular RNAs (circRNAs) refer to non-coding RNAs which are covalently closed molecules generated from back-splicing [4]. CircRNAs have been reported to play important roles in a variety of cancers and they can act as competing endogenous RNAs (ceRNAs) to regulate messenger RNA (mRNA) expression by sponging microRNA (miRNA) in diverse malignancies. They have also been reported to exert oncogenic or anti-tumor roles in PCa progression. For example, circUCK2 increased TET1 inhibits proliferation and invasion of PCa cells via sponging miRNA-767-5p [5]. Dysregulation of p53-RBM25-mediated circAMOTL1L biogenesis contributes to PCa progression through the circAMOTL1L-miR-193a-5p-Pcdha pathway [6]. Aside from that, circHIPK3 overexpression accelerates the proliferation and invasion of PCa cells through regulating miRNA-338-3p [7]. Referring to a recently published literature [8], we found circSERPINA3 was up-regulated in PCa. However, its specific function and regulatory mechanisms in PCa cells remain unclear. Therefore, we chose to study the role and mechanism of circSERPINA3 in PCa cells.

Cancer cells are reported to rewire cellular metabolism to prompt their growth, survival, and long-term maintenance. This altered metabolism which is featured with increased glucose uptake and transformation of glucose to lactate, is widely known as aerobic glycolysis (the Warburg effect) [9, 10]. Mounting research has demonstrated the promoting role of aerobic glycolysis in multiple cancers. For instance, interaction of PKCε with Smad2/3 contributes to PCa cell proliferation via enhancing aerobic glycolysis [11]. HK2-regulated aerobic glycolysis is required for Pten-/p53-deficiency-driven PCa tumor growth [12]. Moreover, circ_0057553 is proved to facilitate PCa cell malignant behaviors and aerobic glycolysis by targeting YES1 [13]. Hence, the effect of circSERPINA3 on aerobic glycolysis in PCa cells is also worth to be explored.

In this study, we aimed to verify the regulatory role of circSERPINA3 in apoptosis, autophagy and aerobic glycolysis of PCa cells. In addition, its specific mechanisms in PCa cells were also explored. Our study might provide a potential novel target for PCa treatment.

## Methods

### Cell lines and cell culture

Normal prostatic epithelial cell line WPMY-1 and PCa cells (PC-3, DU145 and VCaP) were procured from American Type Culture Collection (ATCC, Manassas, VA, USA). WPMY-1 was cultured in Dulbecco’s Modified Essential Medium (DMEM; Gibco-BRL/Invitrogen, USA) with 5% fetal bovine serum (FBS; Gibco, USA) while PCa cells were cultured in DMEM with 10% FBS. For the luciferase reporter assays in this study, HEK-293T cells were obtained from China Institute for the Control of Pharmaceutical and Biological Products and cultured in DMEM with 10% FBS, 100 U/ml penicillin and 100 g/ml streptomycin (15140122, Gibco-BRL/Invitrogen, USA). Cells mentioned above were all maintained at 37 °C with 5% CO_2_.

### Quantitative reverse transcription polymerase chain reaction (RT-qPCR)

In line with the instruction of TRIzol reagent (Takara, Japan), the isolation of total RNA samples was extracted in PCa cells. RNA integrity number (RIN) was eight. 1 μl of the total RNA was added into 20 μl reaction. Synthesis of complementary DNA (cDNA) for miRNAs was carried out using the One Step miR cDNA Synthesis Kit (D1801, HaiGene, Haerbin, China) and the cDNA for circRNAs and mRNAs was synthesized by PrimeScript RT reagent Kit (TaKaRa, Japan). RT-qPCR reaction for miRNA or circRNAs/mRNAs was achieved with HG miRNA SYBR Green PCR Kit (ZY-61500, HaiGene, Haerbin, China) or SYBR Green PCR Kit (QR0100-1KT, Sigma-Aldrich, USA), followed by 2^−ΔΔCt^ method. β-actin worked as the loading control for circRNA as well as mRNAs, and U6 for miR-653-5p. Primer sequences used in this assay were listed in Table 1.

**Table 1:** Primer sequences used in RT-qPCR assay were listed

Gene name	Primer sequences
circSERPINA3	F:GGACGAGTCGGGAACCAGAG R:CAGGGAATCGCTGTCACCTTC
SERPINA3	F:CCTGAGGCAGAGTTGAGAATG R:TCAGGGGCCTTCAGGACTAA
miR-653-5p	F:CCGAGgtgttgaaacaatct R:TCAACTGGTGTCGTGGA
miR-3164	F:CCGAGtgtgactttaagggaa R:TCAACTGGTGTCGTGGA
miR-6820-3p	F:CCGAGtgtgacttctcccctg R:TCAACTGGTGTCGTGGA
miR-509-3p	F:CCGAGtgattgacatttctg R:TCAACTGGTGTCGTGGA
miR-5586-5p	F:CCGAGtatccagcttgttact R:TCAACTGGTGTCGTGGA
GEMIN2	F:GTCCGCAGTGGAAGAGTTGA R:CAGGTATTCCTGAGGCGTCC
MARCH2	F:AGGGTCTTTGTGGCTTGAGG R:GTGACCTGGGTGACGTACTG
NFIA	F:AGCTCATGGAGCGGCAATAG R:CATCCTGGGTGAGACAGAGC
ADSS	F:CTCGGAGTGGACTTCGGATG R:GCTGCGTTTGCACCTTCTAC
BUD13	F:AAGGCCGAGTATCTGAAGCG R:GCCTCCCAGAAGCTTCCATT
β-actin	F:CTAAGGGGGCGCTCTGTCG R:GCGTAGAGGTCCTTCCTGATG
U6	F:TCCCTTCGGGGACATCCG R:AATTTTGGACCATTTCTCGATTTGT

### Plasmid transfection

For overexpression of circSERPINA3, SERPINA3 and BUD13, the whole sequences were separately synthesized and subcloned into pcDNA3.1 vectors with pcDNA3.1 empty vector as a negative control (NC). For overexpression of miR-653-5p, mimic-miR-653-5p was used. In terms of knockdown of circSERPINA3, SERPINA3 and BUD13, specific shRNAs were respectively designed and established with non-targeting shRNA (sh-NC). MiR-653-5p inhibitor was used to negatively regulate miR-653-5p. The abovementioned plasmids were all procured from GenePharma (Shanghai, China). In line with the supplier’s protocols, transfections were conducted with Lipofectamine 2000 (11668019, Invitrogen, USA). The concentration for used shRNAs and pcDNA3.1 plasmids was 0.8 μg/50 μl. And the concentration for used mimic and inhibitor was 50 nM. Sequences used in this assay were listed in Table 2.

**Table 2:** Sequences for gene down-regulation or up-regulation were displayed

Gene name	Sequences
sh-NC (for circSERPINA3)	5ʹ-CCGGATTTCGTTGGATTGTGCACCACTCGAGTGGTGCACAATCCAACGAAAT-3ʹ
sh-circSERPINA3-1	5ʹ-CCGGATTTCGTTGGATGGTGCACAACTCGAGTTGTGCACCATCCAACGAAATTTTTTG-3ʹ
sh-circSERPINA3-2	5ʹ-CCGGTCGTTGGATGGTGCACAAACTCGAGTTTGTGCACCATCCAACGATTTTTG-3ʹ
sh-circSERPINA3-3	5ʹ-CCGGTAATTTCGTTGGATGGTGCACCTCGAGGTGCACCATCCAACGAAATTATTTTTG-3ʹ
sh-NC (for SERPINA3)	5ʹ-CCGGAGATGACATTCTCAGATTGAACTCGAG TTCAATCTGAGAATGTCATCTTTTTTG-3ʹ
sh-SERPINA3-1	5ʹ-CACCAGAAGATGACATTCTTATCAGCTCGAGCTGATAAGAATGTCATCTTC-3ʹ
sh-SERPINA3-2	5ʹ-CACCGTAAAGAAGATGTAATTCACCACTCGAGTGGTGAATTACATCTTCTTTA-3ʹ
sh-SERPINA3-3	5ʹ-CACCGTCATGAAGAAGATGTTCTGGGCTCGAGCCCAGAACATCTTCTTCATGA-3ʹ
sh-NC (for BUD13)	5ʹ-CCGGACATTGAGCCAAGGGAACTGACTCGAGTCAGTTCCCTTGGCTCAATGTTTTTTG-3ʹ
sh-BUD13-1	5'-CACCACATTAGGAGCCAAATCAGGGCTCGAGCCCTGATTTGGCTCCTAATG-3ʹ
sh-BUD13-2	5ʹ-CACCGTCAGAATCAGAAGATTTGGTCCTCGAGGACCAAATCTTCTGATTCTGA-3'
sh-BUD13-3	5ʹ-CACCATTCTTCTTGATGAAGTTGGCCTCGAGGCCAACTTCATCAAGAAGAA-3ʹ
pcDNA3.1-circSERPINA3	GACGGATCGGGAGATCTCCCGATCCCCTATGGTGCACTCTCAGTACAATCTGCTCTGATGCCGCATAGTTAAGCCAGTATCTGCTCCCTGCTTGTGTGTTGGAGGTCGCTGAGTAGTGCGCGAGCAAAATTTAAGCTACAACAAGGCAAGGCTTGACCGACAATTGCATGAAGAATCTGCTTAGGGTTAGGCGTTTTGCGCTGCTTCGCGATGTACGGGCCAGATATACGCGTTGACATTGATTATTGACTAGTTATTAATAGTAATCAATTACGGGGTCATTAGTTCATAGCCCATATATGGAGTTCCGCGTTACATAACTTACGGTAAATGGCCCGCCTGGCTGACCGCCCAACGACCCCCGCCCATTGACGTCAATAATGACGTATGTTCCCATAGTAACGCCAATAGGGACTTTCCATTGACGTCAATGGGTGGAGTATTTACGGTAAACTGCCCACTTGGCAGTACATCAAGTGTATCATATGCCAAGTACGCCCCCTATTGACGTCAATGACGGTAAATGGCCCGCCTGGCATTATGCCCAGTACATGACCTTATGGGACTTTCCTACTTGGCAGTACATCTACGTATTAGTCATCGCTATTACCATGGTGATGCGGTTTTGGCAGTACATCAATGGGCGTGGATAGCGGTTTGACTCACGGGGATTTCCAAGTCTCCACCCCATTGACGTCAATGGGAGTTTGTTTTGGCACCAAAATCAACGGGACTTTCCAAAATGTCGTAACAACTCCGCCCCATTGACGCAAATGGGCGGTAGGCGTGTACGGTGGGAGGTCTATATAAGCAGAGCTCTCTGGCTAACTAGAGAACCCACTGCTTACTGGCTTATCGAAATTAATACGACTCACTATAGGGAGACCCAAGCTGGCTAGCGTTTAAACTTAAGCTTGGTACCGAGCTCGGATCCACTAGTCCAGTGTGGTGgaattcaaagtgctgagattacaggcgtgagccaccacccccggccCACTTTTTGTAAAGGTACGTACTAATGACTTTTTTTTTATACTTCAGGATATCATCGATACCGGTTTAATTAACACGTGGGTAACCGTTAACCCGCGGAGGTAAGAAGCAAGGAAAAGAATTAggctcggcacggtagctcacacctgtaatcccagcagcggccgcTCGAGTCTAGAGGGCCCGTTTAAACCCGCTGATCAGCCTCGACTGTGCCTTCTAGTTGCCAGCCATCTGTTGTTTGCCCCTCCCCCGTGCCTTCCTTGACCCTGGAAGGTGCCACTCCCACTGTCCTTTCCTAATAAAATGAGGAAATTGCATCGCATTGTCTGAGTAGGTGTCATTCTATTCTGGGGGGTGGGGTGGGGCAGGACAGCAAGGGGGAGGATTGGGAAGACAATAGCAGGCATGCTGGGGATGCGGTGGGCTCTATGGCTTCTGAGGCGGAAAGAACCAGCTGGGGCTCTAGGGGGTATCCCCACGCGCCCTGTAGCGGCGCATTAAGCGCGGCGGGTGTGGTGGTTACGCGCAGCGTGACCGCTACACTTGCCAGCGCCCTAGCGCCCGCTCCTTTCGCTTTCTTCCCTTCCTTTCTCGCCACGTTCGCCGGCTTTCCCCGTCAAGCTCTAAATCGGGGGCTCCCTTTAGGGTTCCGATTTAGTGCTTTACGGCACCTCGACCCCAAAAAACTTGATTAGGGTGATGGTTCACGTAGTGGGCCATCGCCCTGATAGACGGTTTTTCGCCCTTTGACGTTGGAGTCCACGTTCTTTAATAGTGGACTCTTGTTCCAAACTGGAACAACACTCAACCCTATCTCGGTCTATTCTTTTGATTTATAAGGGATTTTGCCGATTTCGGCCTATTGGTTAAAAAATGAGCTGATTTAACAAAAATTTAACGCGAATTAATTCTGTGGAATGTGTGTCAGTTAGGGTGTGGAAAGTCCCCAGGCTCCCCAGCAGGCAGAAGTATGCAAAGCATGCATCTCAATTAGTCAGCAACCAGGTGTGGAAAGTCCCCAGGCTCCCCAGCAGGCAGAAGTATGCAAAGCATGCATCTCAATTAGTCAGCAACCATAGTCCCGCCCCTAACTCCGCCCATCCCGCCCCTAACTCCGCCCAGTTCCGCCCATTCTCCGCCCCATGGCTGACTAATTTTTTTTATTTATGCAGAGGCCGAGGCCGCCTCTGCCTCTGAGCTATTCCAGAAGTAGTGAGGAGGCTTTTTTGGAGGCCTAGGCTTTTGCAAAAAGCTCCCGGGAGCTTGTATATCCATTTTCGGATCTGATCAAGAGACAGGATGAGGATCGTTTCGCATGATTGAACAAGATGGATTGCACGCAGGTTCTCCGGCCGCTTGGGTGGAGAGGCTATTCGGCTATGACTGGGCACAACAGACAATCGGCTGCTCTGATGCCGCCGTGTTCCGGCTGTCAGCGCAGGGGCGCCCGGTTCTTTTTGTCAAGACCGACCTGTCCGGTGCCCTGAATGAACTGCAGGACGAGGCAGCGCGGCTATCGTGGCTGGCCACGACGGGCGTTCCTTGCGCAGCTGTGCTCGACGTTGTCACTGAAGCGGGAAGGGACTGGCTGCTATTGGGCGAAGTGCCGGGGCAGGATCTCCTGTCATCTCACCTTGCTCCTGCCGAGAAAGTATCCATCATGGCTGATGCAATGCGGCGGCTGCATACGCTTGATCCGGCTACCTGCCCATTCGACCACCAAGCGAAACATCGCATCGAGCGAGCACGTACTCGGATGGAAGCCGGTCTTGTCGATCAGGATGATCTGGACGAAGAGCATCAGGGGCTCGCGCCAGCCGAACTGTTCGCCAGGCTCAAGGCGCGCATGCCCGACGGCGAGGATCTCGTCGTGACCCATGGCGATGCCTGCTTGCCGAATATCATGGTGGAAAATGGCCGCTTTTCTGGATTCATCGACTGTGGCCGGCTGGGTGTGGCGGACCGCTATCAGGACATAGCGTTGGCTACCCGTGATATTGCTGAAGAGCTTGGCGGCGAATGGGCTGACCGCTTCCTCGTGCTTTACGGTATCGCCGCTCCCGATTCGCAGCGCATCGCCTTCTATCGCCTTCTTGACGAGTTCTTCTGAGCGGGACTCTGGGGTTCGAAATGACCGACCAAGCGACGCCCAACCTGCCATCACGAGATTTCGATTCCACCGCCGCCTTCTATGAAAGGTTGGGCTTCGGAATCGTTTTCCGGGACGCCGGCTGGATGATCCTCCAGCGCGGGGATCTCATGCTGGAGTTCTTCGCCCACCCCAACTTGTTTATTGCAGCTTATAATGGTTACAAATAAAGCAATAGCATCACAAATTTCACAAATAAAGCATTTTTTTCACTGCATTCTAGTTGTGGTTTGTCCAAACTCATCAATGTATCTTATCATGTCTGTATACCGTCGACCTCTAGCTAGAGCTTGGCGTAATCATGGTCATAGCTGTTTCCTGTGTGAAATTGTTATCCGCTCACAATTCCACACAACATACGAGCCGGAAGCATAAAGTGTAAAGCCTGGGGTGCCTAATGAGTGAGCTAACTCACATTAATTGCGTTGCGCTCACTGCCCGCTTTCCAGTCGGGAAACCTGTCGTGCCAGCTGCATTAATGAATCGGCCAACGCGCGGGGAGAGGCGGTTTGCGTATTGGGCGCTCTTCCGCTTCCTCGCTCACTGACTCGCTGCGCTCGGTCGTTCGGCTGCGGCGAGCGGTATCAGCTCACTCAAAGGCGGTAATACGGTTATCCACAGAATCAGGGGATAACGCAGGAAAGAACATGTGAGCAAAAGGCCAGCAAAAGGCCAGGAACCGTAAAAAGGCCGCGTTGCTGGCGTTTTTCCATAGGCTCCGCCCCCCTGACGAGCATCACAAAAATCGACGCTCAAGTCAGAGGTGGCGAAACCCGACAGGACTATAAAGATACCAGGCGTTTCCCCCTGGAAGCTCCCTCGTGCGCTCTCCTGTTCCGACCCTGCCGCTTACCGGATACCTGTCCGCCTTTCTCCCTTCGGGAAGCGTGGCGCTTTCTCATAGCTCACGCTGTAGGTATCTCAGTTCGGTGTAGGTCGTTCGCTCCAAGCTGGGCTGTGTGCACGAACCCCCCGTTCAGCCCGACCGCTGCGCCTTATCCGGTAACTATCGTCTTGAGTCCAACCCGGTAAGACACGACTTATCGCCACTGGCAGCAGCCACTGGTAACAGGATTAGCAGAGCGAGGTATGTAGGCGGTGCTACAGAGTTCTTGAAGTGGTGGCCTAACTACGGCTACACTAGAAGAACAGTATTTGGTATCTGCGCTCTGCTGAAGCCAGTTACCTTCGGAAAAAGAGTTGGTAGCTCTTGATCCGGCAAACAAACCACCGCTGGTAGCGGTTTTTTTGTTTGCAAGCAGCAGATTACGCGCAGAAAAAAAGGATCTCAAGAAGATCCTTTGATCTTTTCTACGGGGTCTGACGCTCAGTGGAACGAAAACTCACGTTAAGGGATTTTGGTCATGAGATTATCAAAAAGGATCTTCACCTAGATCCTTTTAAATTAAAAATGAAGTTTTAAATCAATCTAAAGTATATATGAGTAAACTTGGTCTGACAGTTACCAATGCTTAATCAGTGAGGCACCTATCTCAGCGATCTGTCTATTTCGTTCATCCATAGTTGCCTGACTCCCCGTCGTGTAGATAACTACGATACGGGAGGGCTTACCATCTGGCCCCAGTGCTGCAATGATACCGCGAGACCCACGCTCACCGGCTCCAGATTTATCAGCAATAAACCAGCCAGCCGGAAGGGCCGAGCGCAGAAGTGGTCCTGCAACTTTATCCGCCTCCATCCAGTCTATTAATTGTTGCCGGGAAGCTAGAGTAAGTAGTTCGCCAGTTAATAGTTTGCGCAACGTTGTTGCCATTGCTACAGGCATCGTGGTGTCACGCTCGTCGTTTGGTATGGCTTCATTCAGCTCCGGTTCCCAACGATCAAGGCGAGTTACATGATCCCCCATGTTGTGCAAAAAAGCGGTTAGCTCCTTCGGTCCTCCGATCGTTGTCAGAAGTAAGTTGGCCGCAGTGTTATCACTCATGGTTATGGCAGCACTGCATAATTCTCTTACTGTCATGCCATCCGTAAGATGCTTTTCTGTGACTGGTGAGTACTCAACCAAGTCATTCTGAGAATAGTGTATGCGGCGACCGAGTTGCTCTTGCCCGGCGTCAATACGGGATAATACCGCGCCACATAGCAGAACTTTAAAAGTGCTCATCATTGGAAAACGTTCTTCGGGGCGAAAACTCTCAAGGATCTTACCGCTGTTGAGATCCAGTTCGATGTAACCCACTCGTGCACCCAACTGATCTTCAGCATCTTTTACTTTCACCAGCGTTTCTGGGTGAGCAAAAACAGGAAGGCAAAATGCCGCAAAAAAGGGAATAAGGGCGACACGGAAATGTTGAATACTCATACTCTTCCTTTTTCAATATTATTGAAGCATTTATCAGGGTTATTGTCTCATGAGCGGATACATATTTGAATGTATTTAGAAAAATAAACAAATAGGGGTTCCGCGCACATTTCCCCGAAAAGTGCCACCTGACGTC
pcDNA3.1-BUD13	atggcggcagctccgccgctttccaaggccgagtatctgaagcgttacttgtccggggcagatgccggcgtcgaccggggatctgagtccggtcgcaagcgtcgcaaaaagcggccgaagcctggcggggccggcggcaagggaatgcggattgtggatgatgatgtgagctggacagctatctccacaaccaaactagaaaaggaggaagaggaagatgatggagatttgcctgtggtggcagagtttgtggatgagcggccagaagaggtaaagcagatggaggcctttcgttccagtgccaaatggaagcttctgggaggccacaacgaagacctaccctcaaacagacattttcgtcacgataccccggattcatctcctaggagggtccgtcatggtaccccagatccatctcctaggaaggaccgtcatgacaccccggatccatctcctaggagggcccgtcatgacaccccggatccttctcccctcagaggggctcgtcatgactcagacacatctcctcccaggaggatccgtcatgactcctcagacacttcacccccaaggagggcccgtcatgattctccagatccttctcccccaaggaggcctcagcataattcttcaggtgactgccagaaagcaactgattcagacctttcttctccacggcataaacaaagtccagggcaccaggattctgattcagatctgtcacctccacggaatagacctagacaccggagctctgattctgacctctctccaccaaggaggagacagaggaccaaatcttctgattctgacctgtccccgcctcgaaggagtcagcctcctggaaagaaggctgcacacatgtattctggggctaaaactgggttggtgttaactgacatacagcgagaacagcaggagctcaaggaacaggatcaagaaaccatggcatttgaagctgaatttcaatatgctgaaaccgtatttcgagataagtctggtcgtaagaggaatttgaaactcgaacgtttagagcaaaggaggaaagcagaaaaggactcagagagagatgagctgtatgcccagtggggaaaagggcttgcccagagccggcaacagcaacaaaatgtggaggatgcaatgaaagagatgcaaaagcctctggcccgctatattgatgacgaagatctggataggatgctaagagaacaggaaagagagggggaccctatggccaacttcatcaagaagaataaggccaaggagaacaagaataaaaaagtgagacctcgctacagtggtccagcacctcctcccaacagatttaatatctggcctggatatcgctgggacggagtggacagatccaatggatttgaacagaagcgctttgccaggcttgccagcaagaaggcagtggaggaacttgcctacaaatggagtgttgaggatatgtaa
pcDNA3.1-SERPINA3	atggagagaatgttacctctcctggctctggggctcttggcggctgggttctgccctgctgtcctctgccaccctaacagcccacttgacgaggagaatctgacccaggagaaccaagaccgagggacacacgtggacctcggattagcctccgccaacgtggacttcgctttcagcctgtacaagcagttagtcctgaaggcccctgataagaatgtcatcttctccccactgagcatctccaccgccttggccttcctgtctctgggggcccataataccaccctgacagagattctcaaaggcctcaagttcaacctcacggagacttctgaggcagaaattcaccagagcttccagcacctcctgcgcaccctcaatcagtccagcgatgagctgcagctgagtatgggaaatgccatgtttgtcaaagagcaactcagtctgctggacaggttcacggaggatgccaagaggctgtatggctccgaggcctttgccactgactttcaggactcagctgcagctaagaagctcatcaacgactacgtgaagaatggaactagggggaaaatcacagatctgatcaaggaccttgactcgcagacaatgatggtcctggtgaattacatcttctttaaagccaaatgggagatgccctttgacccccaagatactcatcagtcaaggttctacttgagcaagaaaaagtgggtaatggtgcccatgatgagtttgcatcacctgactataccttacttccgggacgaggagctgtcctgcaccgtggtggagctgaagtacacaggcaatgccagcgcactcttcatcctccctgatcaagacaagatggaggaagtggaagccatgctgctcccagagaccctgaagcggtggagagactctctggagttcagagagataggtgagctctacctgccaaagttttccatctcgagggactataacctgaacgacatacttctccagctgggcattgaggaagccttcaccagcaaggctgacctgtcagggatcacaggggccaggaacctagcagtctcccaggtggtccataaggctgtgcttgatgtatttgaggagggcacagaagcatctgctgccacagcagtcaaaatcaccctcctttctgcattagtggagacaaggaccattgtgcgtttcaacaggcccttcctgatgatcattgtccctacagacacccagaacatcttcttcatgagcaaagtcaccaatcccaagcaagcctag
mimic-NC	tgtaaacaatctactgctgtg
mimic-miR-653-5p	guguugaaacaaucucuacug
miR-653-5p inhibitor	aguagagauuguuucaacac

### Fluorescence in situ hybridization (FISH)

In line with RiboTM Fluorescent In Situ Hybridization Kit (Ribobio, Guangzhou, China), the FISH-probe designed for circSERPINA3 was used as per direction. Nuclei were counterstained with DAPI for observation by using confocal laser scanning microscope (Smart zoom 5, Zeiss). The fluorescence intensity was quantified by Image J. The overlap of green fluorescence and DAPI staining represented distribution of the cell nucleus. The rest part represented cytoplasmic distribution. The subcellular localization of circSERPINA3 in DU145 cells was determined.

### Subcellular fractionation assay

After the cytoplasmic and nuclear parts of PCa cells were separated by centrifugation, the expression of circSERPINA3 in different fragments was quantified by RT-qPCR. U6 or β-actin served as control in nucleus or cytoplasm respectively.

### Flow cytometry for apoptotic cells

Annexin V-FITC Apoptosis Detection Kit (Beyotime, USA) was utilized to monitor the apoptotic cells based on the user manual. Transfected PCa cells were collected from pre-cooled PBS for dual-staining and then analyzed by flow cytometer (BD Biosciences, Franklin Lakes, NJ, USA). The formula for calculating cell apoptosis was Early and later apoptotic cell number/Total cell number.

### RNA pull down assay

To verify the binding capacity between circSERPINA3 and miR-653-5p, circSERPINA3 with wild or mutant binding sites was labeled with the biotin. Cell lysates were mixed with bio-NC, bio-circSERPINA3-WT or bio-circSERPINA3-Mut for 1 h. After that, RNAs were purified with TRIzol reagent (Takara, Japan). The enrichment of miR-653-5p was analyzed by RT-qPCR. Likewise, SERPINA3 with wild or mutant sequences was labeled with the biotin and the enrichment of miR-653-5p was analyzed by RT-qPCR accordingly to verify the binding capacity between SERPINA3 and miR-653-5p.

### RNA immunoprecipitation (RIP) assay

With the ProteoPrep® Total Extraction Sample Kit (PROTTOT-1KT, Sigma-Aldrich, USA), RIP assays in PC-3 cells were achieved with the specific antibodies including Anti-BUD13 (PA5-58351, Invitrogen), Anti-HNRNPA1 (ab177152, Abcam, Cambridge, MA, USA), Anti-U2AF2 (ab197031, Abcam), Anti-PCBP2 (ab184962), Anti-FAM120A (ab229254, Abcam), Anti-GTF2F1 (AV100810, Sigma-Aldrich, St. Louis, MO, USA), Anti-BCCIP (ab168157, Abcam) and negative control anti-IgG antibody (ab133470, Abcam). Lysates were obtained with the help of RIP lysis buffer (KeyGEN Biotech, Nanjing, China), and the lysis was incubated with the magnetic beads conjugated with the IgG antibody. In this study, the specific antibodies of potential RNA binding protein (RBP) were involved and RT-qPCR was used to examine the enrichment of circSERPINA3 and SERPINA3 among the candidate RBP.

### Dual-luciferase reporter assay

For luciferase reporter assay, full-length sequence of circSERPINA3 (443 bp) or SERPINA3 3ʹUTR (239 bp) possessing wild-type (WT) and mutant (Mut) miR-653-5p binding sites were sub-cloned into pmirGLO luciferase vectors to obtain pmirGLO-circSERPINA3-WT/Mut and pmirGLO-SERPINA3 3ʹUTR-WT/Mut. The sequence of circSERPINA3/SERPINA3 3ʹUTR-Mut was obtained by mutating the complementary bases of miR-653-5p on circSERPINA3/SERPINA3 3ʹUTR-WT. MiR-653-5p mimics or NC mimics were co-transfected with circSERPINA3-WT/Mut or SERPINA3 3ʹUTR-WT/Mut into PCa cells. After 48-h transfection, cells were extracted and the luciferase activities were analyzed utilizing the dual-luciferase reporter assay system (Promega).

### Western blot

Total protein extracted from PCa cell lines was isolated by RIPA buffer, and after being separated through sodium dodecyl sulfate polyacrylamide gel electrophoresis (SDS-PAGE), proteins were transferred to polyvinylidene fluoride (PVDF) membranes. 5% skim milk was then used for blocking the membranes. The membranes were incubated with primary antibodies including Anti-LC3 (ab192890, Abcam), Anti-p62 (ab109012, Abcam), Anti-BUD13 (PA5-58351, Invitrogen), Anti-β-actin (ab179467, Abcam), Anti-Bcl-2 (ab32124, Abcam), Anti-Bax (ab32503, Abcam), Anti-cleaved PARP1 (ab32064, Abcam) overnight at 4 °C, followed by being cultivated with secondary antibody for 1 h. After washing in TBST, the secondary antibodies (ab7090, Abcam) were added, finally assayed by enhanced chemiluminescence (ECL) detection system.

### Caspase3 activity assay

Caspase3 activity assay was achieved using Caspase3 Activity Assay Kit as required by supplier (KeyGEN Biotech, Nanjing, China). The total protein of cells was obtained through lysis buffer for incubation with reaction buffer and caspase substrate. Caspase3 activity was measured at wavelength of 405 nm with the help of microplate reader.

### Caspase9 activity assay

Caspase9 activity assay was achieved with Caspase9 Activity Assay Kit (KeyGEN Biotech, Nanjing, China). Protein extracts were incubated with caspase9 substrate in provided reaction buffer. The absorbance of Caspase9 activity was measured at 405 nm under the microplate reader. Relative caspase activity was calculated as a ratio of the emission of treated cells to untreated cells.

### Statistical analysis

All data from experiments were exhibited as the mean ± standard deviation (SD). Data analysis was achieved by using SPSS 17.0 software. Student’s *t* test was used for evaluation the difference between two groups and one-way/two-way ANOVA for evaluation among multiple groups with Tukey and Dunnett as the back testing methods. Statistics with p value below 0.05 were considered to be statistically significant.

## Results

### CircSERPINA3 inhibits PCa cell apoptosis while promoting cell autophagy and aerobic glycolysis

**Fig. 1:**
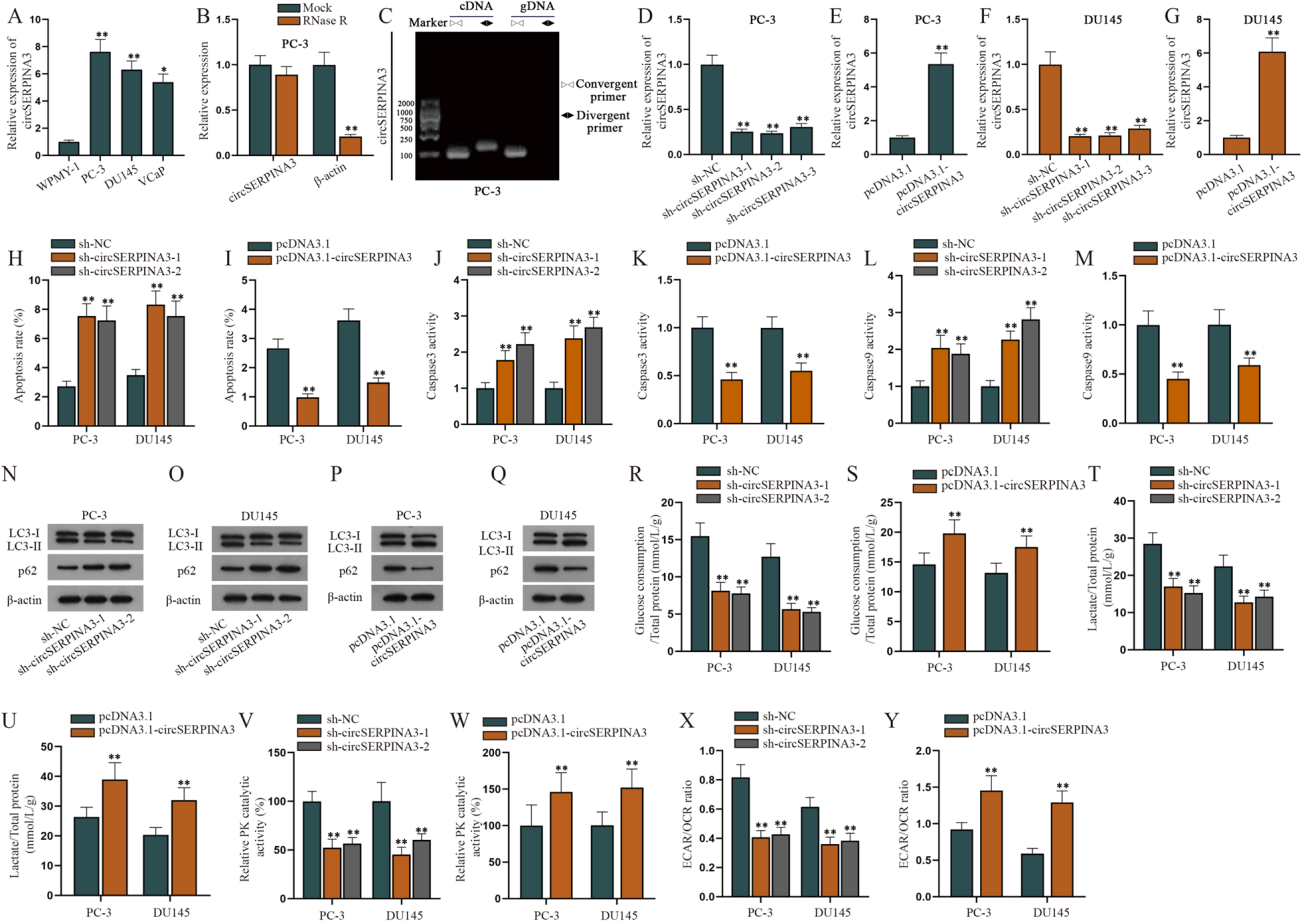
circSERPINA3 inhibits cell apoptosis while promoting autophagy and aerobic glycolysis in PCa cells. **A** RT-qPCR was utilized to examine circSERPINA3 expression in normal WPMY-1 cell line and PCa cells (PC-3, DU145 and VCaP). **B** The expression level of circSERPINA3 after RNase R treatment was examined by RT-qPCR. **C** AGE-PCR was used to verify the closed-loop structure of circSERPINA3 in PC-3 cells. **D** The knockdown efficiency of sh-circSERPINA3-1/2/3 in PC-3 cells was examined via RT-qPCR. **E** The overexpression efficiency of pcDNA3.1-circSERPINA3 in PC-3 cells was tested by means of RT-qPCR. **F** The expression of circSERPINA3 was detected via RT-qPCR after DU145 cells were transfected with sh-circSERPINA3-1/2/3. **G** The expression of circSERPINA3 was quantified via RT-qPCR after DU145 cells were transfected with pcDNA3.1-circSERPINA3. **H**, **I** An analysis of annexin V staining by flow cytometry was applied to test the apoptosis rate of PCa cells upon circSERPINA3 depletion or overexpression. **J**, **K** Caspase3 Activity Assay Kit was applied to examine Caspase3 activity when circSERPINA3 was cut down in PCa cells. **L**, **M** In response to circSERPINA3 knockdown or augment, Caspase9 activity in PCa cells was detected by Caspase9 Activity Assay Kit. **N**, **O** Western blot analysis was performed to examine the protein levels of LC3-I, LC3-II and p62 in PCa cells after circSERPINA3 down-regulation. **P**, **Q** The protein levels of LC3-I, LC3-II and p62 were detected by western blot after circSERPINA3 augment in PCa cells. **R**, **S** Glucose consumption in PCa cells was examined after circSERPINA3 down-regulation or overexpression. **T**, **U** Lactate content was detected in PCa cells transfected with sh-circSERPINA3-1/2 or pcDNA3.1-circSERPINA3. **V**, **W** PK activity was examined in PCa cells with circSERPINA3 knockdown or up-regulation. **X**, **Y** ECAR/OCR value in PCa cells was tested under the condition of circSERPINA3 depletion or overexpression. (*cDNA* complementary DNA, *gDNA* genomic DNA, *sh* short hairpin, *NC* negative control). ^*^P < 0.05, ^**^P < 0.01

At first, we examined the expression level of circSERPINA3 in normal prostatic epithelial cell line WPMY-1 and PCa cells comprising PC-3, DU145 and VCaP. According to RT-qPCR analysis, the expression of circSERPINA3 was higher in PCa cells than that in WPMY-1 cells (Fig. 1A), and PC-3 and DU145 cells were chosen for further studies. Based on the fact that linear RNA was likely to degrade while circRNA is relatively stable [14], we treated circSERPINA3 and the reference gene β-actin with RNase R. According to the data from RT-qPCR, no obvious change was observed in circSERPINA3 expression, while β-actin was overtly degraded (Fig. 1B). Therefore, the circular property of circSERPINA3 was verified. Agarose gel electrophoresis (AGE) with PCR was then used for further verification (Fig. 1C). It turned out cDNA of circSERPINA3 could be detected by both divergent and convergent primers, re-confirming its circular structure. Next, the knockdown efficiency of sh-circSERPINA3-1/2/3 in PCa cells as well as the overexpression efficiency of pcDNA3.1-circSERPINA3 in PCa cells and WPMY-1 cells were determined via RT-qPCR (Fig. 1D–G and Additional file 1: Figure S1A). The results indicated that circSERPINA3 was obviously down-regulated or up-regulated after the transfection of abovementioned plasmids. In addition, sh-circSERPINA3-1 and sh-circSERPINA3-2 were involved in the subsequent assays for their higher knockdown efficiency than sh-circSERPINA3-3. After cells were transfected for 48 h, an analysis of annexin V staining by flow cytometry was applied to test the apoptosis rate of PCa cells upon circSERPINA3 up-regulation and down-regulation (Fig. 1H, I). The results indicated that apoptosis rate of PCa cells rose after circSERPINA3 reduction and declined after circSERPINA3 augment. Moreover, apoptosis rate was discovered to be diminished in WPMY-1 cells after circSERPINA3 was overexpressed (Additional file 1: Figure S1B). Since Caspase3 and Caspase9 were apoptosis-related proteins and their high expression suggested high apoptosis degree [15], we examined Caspase3 and Caspase9 activity when circSERPINA3 was reduced or overexpressed in PCa cells (Fig. 1J–M). Results showed that Caspase3 and Caspase9 activity enhanced when circSERPINA3 was inhibited, while dropping when circSERPINA3 was overexpressed in PCa cells. In addition, Caspase3 and Caspase9 activities were also detected in WPMY-1 cells transfected with pcDNA3.1-circSERPINA3. The obtained data indicated that Caspase3 and Caspase9 activities declined due to circSERPINA3 elevation (Additional file 1: Figure S1C, D). To reconfirm circSERPINA3 could regulate PCa cell and WPMY-1 cell apoptosis, the expression of apoptosis-related proteins was examined by western blot. It turned out circSERPINA3 knockdown led to reduced level of Bcl-2 and elevated levels of Bax and Cleaved-PARP1 in PCa cells, while circSERPINA3 overexpression resulted in opposite results in PCa cells and WPMY-1 cells (Additional file 1: Figure S1E–I). Therefore, we concluded that circSERPINA3 negatively regulated PCa cell and WPMY-1 cell apoptosis. Next, we tried to explore whether circSERPINA3 could affect cell autophagy. Referring to relative documents, we learned that decreased p62 expression with the transition from LC3-I to LC3-II could be seen as a mark of autophagy [16]. Therefore, we carried out western blot analysis to examine the expression of LC3-I, LC3-II and p62 in PCa cells transfected with sh-circSERPINA3 or pcDNA3.1-circSERPINA3 (Fig. 1N–Q). Results showed that when circSERPINA3 was down-regulated in PCa cells, the expression of p62 and LC3-I increased, while LC3-II decreased, which showed that circSERPINA3 positively regulated PCa cell autophagy. To further prove the impact of circSERPINA3 on cell autophagy, Rapamycin, an activator of cell autophagy [17], was used in the following rescue assays. As shown in western blot assays, PCa cell autophagy was enhanced after cells were treated with Rapamycin and was inhibited again by circSERPINA3 reduction (Additional file 1: Figure S1J–K). Furthermore, it was reported that the higher glucose consumption was, the higher aerobic glycolysis degree was [18]. In this study, we examined the glucose consumption in PCa cells and found that the glucose consumption was decreased due to circSERPINA3 down-regulation and increased by circSERPINA3 elevation (Fig. 1R–S). Moreover, we tested lactate content, pyruvate kinase (PK) activity and extracellular acidification rate/oxygen consumption rate (ECAR/OCR) value in PCa cells. We found that circSERPINA3 knockdown led to decrease in lactate content, while circSERPINA3 overexpression made the contrary difference (Fig. 1T, U). Meanwhile, the change of PK activity and ECAR/OCR value was consistent with that of lactate content in sh-circSERPINA3-1/2 or pcDNA3.1-circSERPINA3 transfected PCa cells (Fig. 1V–Y), which proved that circSERPINA3 positively regulated aerobic glycolysis in PCa cells. Based on the results from Additional file 1: Figure S1L-O, circSERPINA3 could also promote aerobic glycolysis in WPMY-1 cells as the changes of aerobic glycolysis-related factors in circSERPINA3-elevated WPMY-1 cells was in line with those in circSERPINA3-elevated PCa cells. Taken together, circSERPINA3 represses cell apoptosis, while promoting autophagy and aerobic glycolysis in PCa cells.

### CircSERPINA3 regulates SERPINA3 through competitively binding to miR-653-5p

**Fig. 2:**
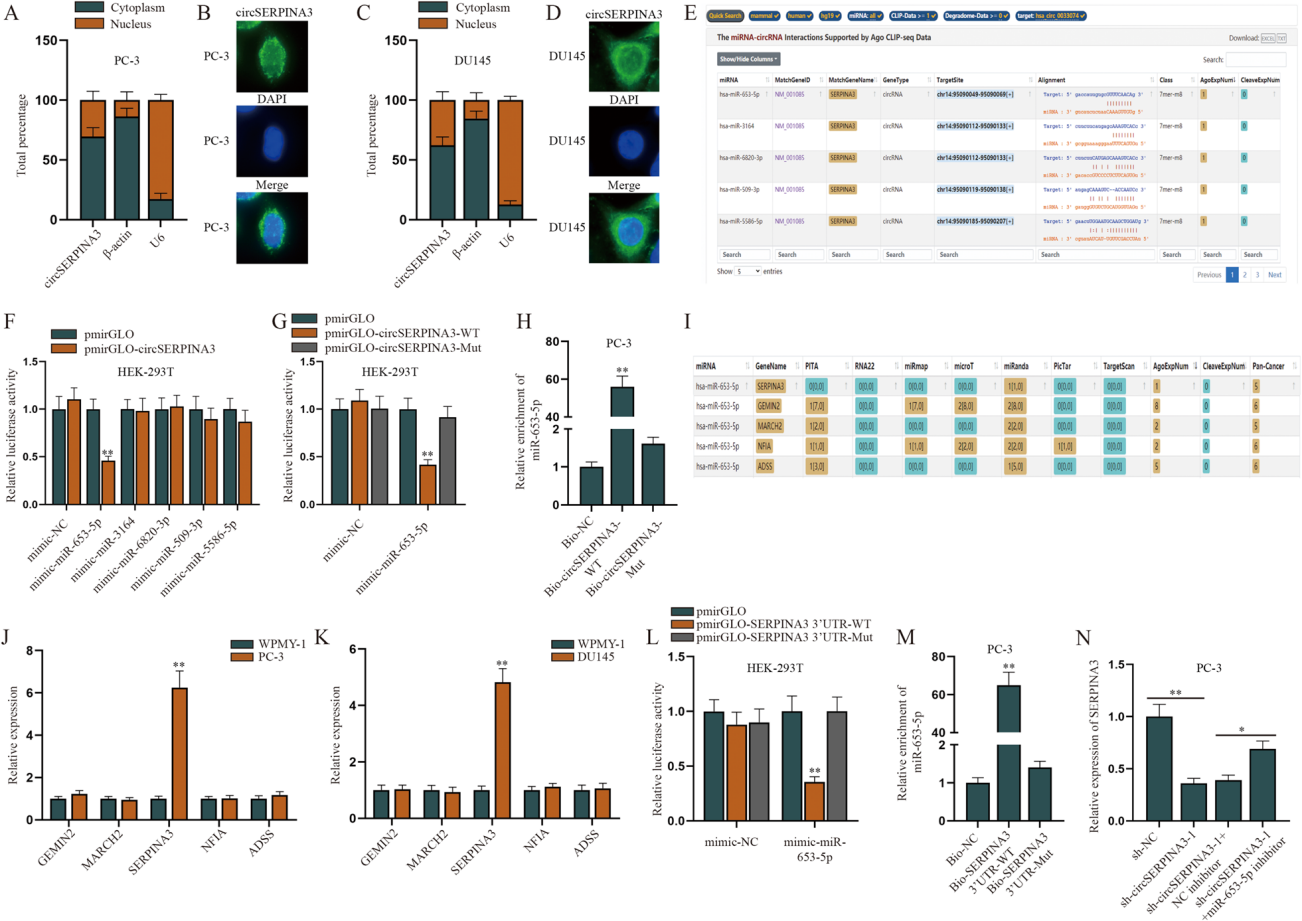
CircSERPINA3 regulates SERPINA3 through competitively binding to miR-653-5p. **A**, **B** Subcellular fractionation assay and FISH assay were conducted to identify the location of circSERPINA3 in PC-3 cells. **C**, **D** Subcellular fractionation assay and FISH assay were implemented to examine the distribution of circSERPINA3 in DU145 cells. **E** Potential miRNAs that could bind to circSERPINA3 were selected with the help of starBase. **F** Potential five miRNAs were selected and respectively overexpressed in pmirGLO-circSERPINA3 transfected HEK-293T cells and then dual luciferase reporter assays were conducted to detect the luciferase activity of pmirGLO-circSERPINA3 plasmids. **G** Dual luciferase reporter assay was carried out to verify the binding capacity between miR-653-5p and circSERPINA3 based on the luciferase activity of pmirGLO-circSERPINA3-WT/Mut vectors. **H** RNA pull down assay was implemented to evaluate the enrichment of miR-653-5p in Bio-circSERPINA3-WT/Mut. **I** Five downstream mRNAs for miR-653-5p was predicted through starBase. **J**, **K** RT-qPCR was utilized to examine the expression of the potential mRNAs in normal WPMY-1 cells and PCa cells. **L** The luciferase activity of pmirGLO-SERPINA3 3’UTR-WT/Mut was detected after mimic-miR-653-5p transfection by means of dual luciferase reporter assay to verify the binding capacity between miR-653-5p and SERPINA3. **M** RNA pull down assay was done to test the enrichment of miR-653-5p in Bio-SERPINA3 3ʹUTR-WT/Mut. **N** After PC-3 cells were transfected with sh-NC, sh-circSERPINA3-1, sh-circSERPINA3-1 + NC inhibitor or sh-circSERPINA3-1 + miR-653-5p inhibitor, a series of recue assays were conducted to detect the expression of SERPINA3. (*NC* negative control, *WT* wide-type, *Mut* Mutant, *Bio* biotinylated, *sh* short hairpin). ^*^P < 0.05, ^**^P < 0.01

For the next-step study, we explored the regulatory pattern of circSERPINA3 in PCa cells. First of all, we used subcellular fractionation assay and FISH assay to identify the location of circSERPINA3 in PC-3 and DU145 cells, and it was shown that circSERPINA3 was located in both cytoplasm and nucleus of PCa cells (Fig. 2A–D). Based on previous studies, when circRNAs were located in cytoplasm, they were likely to sponge miRNAs and then exert their functions by regulating expression of its host gene [19]. Therefore, we selected potential miRNAs that might bind to circSERPINA3 with the help of starBase (http://starbase.sysu.edu.cn/) under the condition of CLIP-Data ≥ 1, and the first five miRNAs were chosen for the follow-up experiments (Fig. 2E). Dual luciferase reporter assays were then conducted when HEK-293T cells were transfected with mimic-miRNA of the above 5 miRNA candidates and circSERPINA3-WT. Results showed that the luciferase activity of pmirGLO-circSERPINA3 displayed an obvious decline only when miR-653-5p-mimic was transfected into HEK-293T cells (Fig. 2F). Later, we conducted dual luciferase reporter assay and RNA pull down analysis to further verify the binding affinity between miR-653-5p and circSERPINA3. The outcomes of luciferase reporter assay manifested that luciferase activity of pmirGLO-circSERPINA3-WT was overtly weakened in HEK-293T cells transfected with mimic-miR-653-5p while that of pmirGLO-circSERPINA3-Mut had no obvious change (Fig. 2G). And data obtained from RNA pull down assay indicated that miR-653-5p was largely enriched in Bio-circSERPINA3-WT rather than Bio-circSERPINA3-Mut (Fig. 2H). After identifying the target miRNA, we used starBase again to predict the downstream mRNAs for miR-653-5p (selection conditions: CLIP-Data ≥ 1, Pan-Cancer ≥ 5) (Fig. 2I). RT-qPCR was firstly utilized to examine the expression of the five mRNAs in normal WPMY-1 cells and PCa cells and it was displayed that only SERPINA3 was up-regulated in PCa cells (Fig. 2J, K). Therefore, we chose SERPINA3 and examine its binding relationship with miR-653-5p. Dual luciferase reporter assays were performed and pmirGLO-SERPINA3 3’UTR-WT/Mut was co-transfected with mimic-miR-653-5p or mimic-NC. As presented in Fig. 2L, the luciferase activity of pmirGLO-SERPINA3 3ʹUTR-WT displayed a sharp decrease, whereas no obvious change was seen in its mutant type. RNA pull down assay further demonstrated that miR-653-5p could bind to SERPINA3 (Fig. 2M). Finally, the rescue experiment were conducted to verify the relationship among SERPINA3, miR-653-5p and circSERPINA3 (Fig. 2N). RT-qPCR results implied that miR-653-5p down-regulation could attenuate but not completely reverse the inhibitory effect of sh-circSERPINA3-1 on SERPINA3. Therefore, we predicted that circSERPINA3 may regulate SERPINA3 through other ways. Recent studies have pointed out that circRNAs could work as RNA binding protein (RBP) sponges to perform their multiple functions [20]. Accordingly, we carried out the next-step experiments to identify the certain RBP for circSERPINA3.

### CircSERPINA3 regulates SERPINA3 mRNA stability via recruiting BUD13

**Fig. 3:**
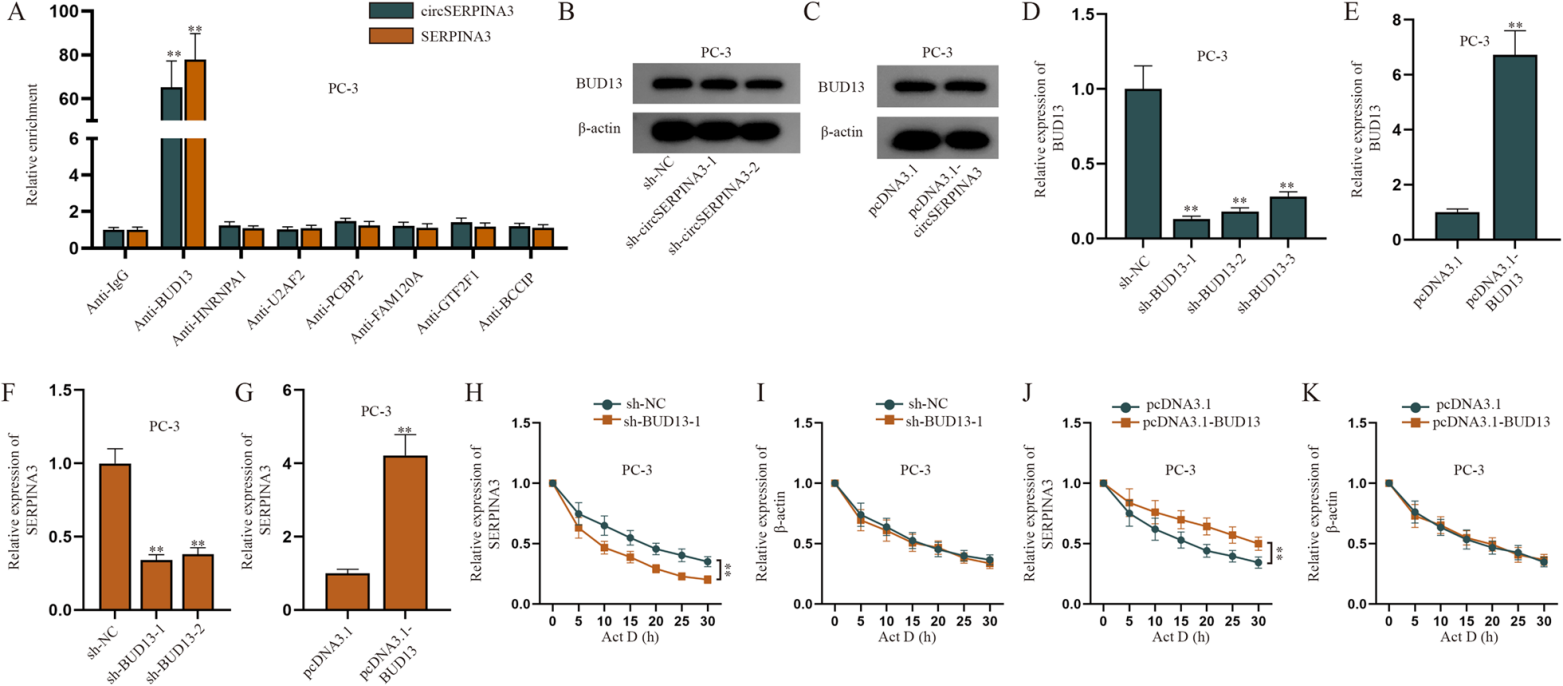
CircSERPINA3 stabilizes SERPINA3 via recruiting BUD13. **A** RIP assay was used to examine the enrichment of circSERPINA3 and SERPINA3 in PC-3 cells incubated with different antibodies of candidate shared RBP screened out from starBase. **B**, **C** Western blot analysis was conducted to examine BUD13 expression when circSERPINA3 was down-regulated or overexpressed in PC-3 cells. **D**, **E** RT-qPCR was used to examine the knockdown efficiency of sh-BUD13-1/2/3 and overexpression efficiency of pcDNA3.1-BUD13 in PCa cells. **F**, **G** RT-qPCR was carried out to examine SERPINA3 expression when BUD13 was reduced or overexpressed in PC-3 cells. **H**, **I** RT-qPCR was used to examine the effect of sh-BUD13-1 on half-life of SERPINA3 and β-actin in PC-3 cells. **J**, **K** Expression of SERPINA3 and β-actin in PC-3 cells was tested by means of RT-qPCR after cells were transfected with pcDNA3.1-BUD13. (*sh* short hairpin, *NC* negative control). ^**^P < 0.01

Firstly, we utilized starBase to predict potential RBP possibly binding to circSERPINA3 with the condition of: CLIP-Data ≥ 2 and ClusterNum ≥ 15 and the candidate RBP likely binding with SERPINA3 with the condition of CLIP-Data ≥ 2 and ClusterNum ≥ 15. As shown in Additional file 2: Figure S2A, the potential common RBP was screened out, including HNRNP1, U2AF2, PCBP2 BCCIP, FAM120A, GTF2F1 and BUD13. Then, we examined the expression of circSERPINA3 and SERPINA3 enriched in the corresponding antibodies of these candidate shared RBP in PC-3 cells (Fig. 3A). It turned out shown that circSERPINA3 and SERPINA3 could largely bind to BUD13. Western blot analysis was then conducted to examine BUD13 expression when circSERPINA3 was diminished or overexpressed in PC-3 cells (Fig. 3B, C), and results showed that circSERPINA3 didn’t affect the expression of BUD13. Furthermore, RT-qPCR was used to examine the interference of sh-BUD13-1/2/3 and the overexpression efficiency of pcDNA3.1-BUD13 in PC-3 cells (Fig. 3D, E). It turned out BUD13 was dramatically cut down by sh-BUD13-1/2/3 and elevated by pcDNA3.1-BUD13. Sh-BUD13-1 and sh-BUD13-2 were chosen for the further studies due to their higher efficiency than sh-BUD13-3. Afterwards, RT-qPCR was carried out to examine SERPINA3 expression when BUD13 was decreased or overexpressed in PC-3 cells (Fig. 3F, G), and results showed that the expression of SERPINA3 was decreased by BUD13 down-regulation and raised by BUD13 up-regulation, which demonstrated that BUD13 could positively regulate SERPINA3. Since we learned from previous literature that BUD13 could exert its function in tumor through regulating mRNA stability [21], we wanted to explore whether BUD13 could regulate the mRNA stability of SERPINA3. Therefore, we examined the effect of BUD13 knockdown on SERPINA3 mRNA half-life (Fig. 3H–K), and noticed that the half-life of SERPINA3 mRNA was obviously shortened due to BUD13 down-regulation and prolonged after BUD13 overexpression. Taken together, circSERPINA3 regulates SERPINA3 mRNA stability via recruiting BUD13.

### SERPINA3 inhibits cell apoptosis while promoting autophagy and aerobic glycolysis in PCa cells

**Fig. 4:**
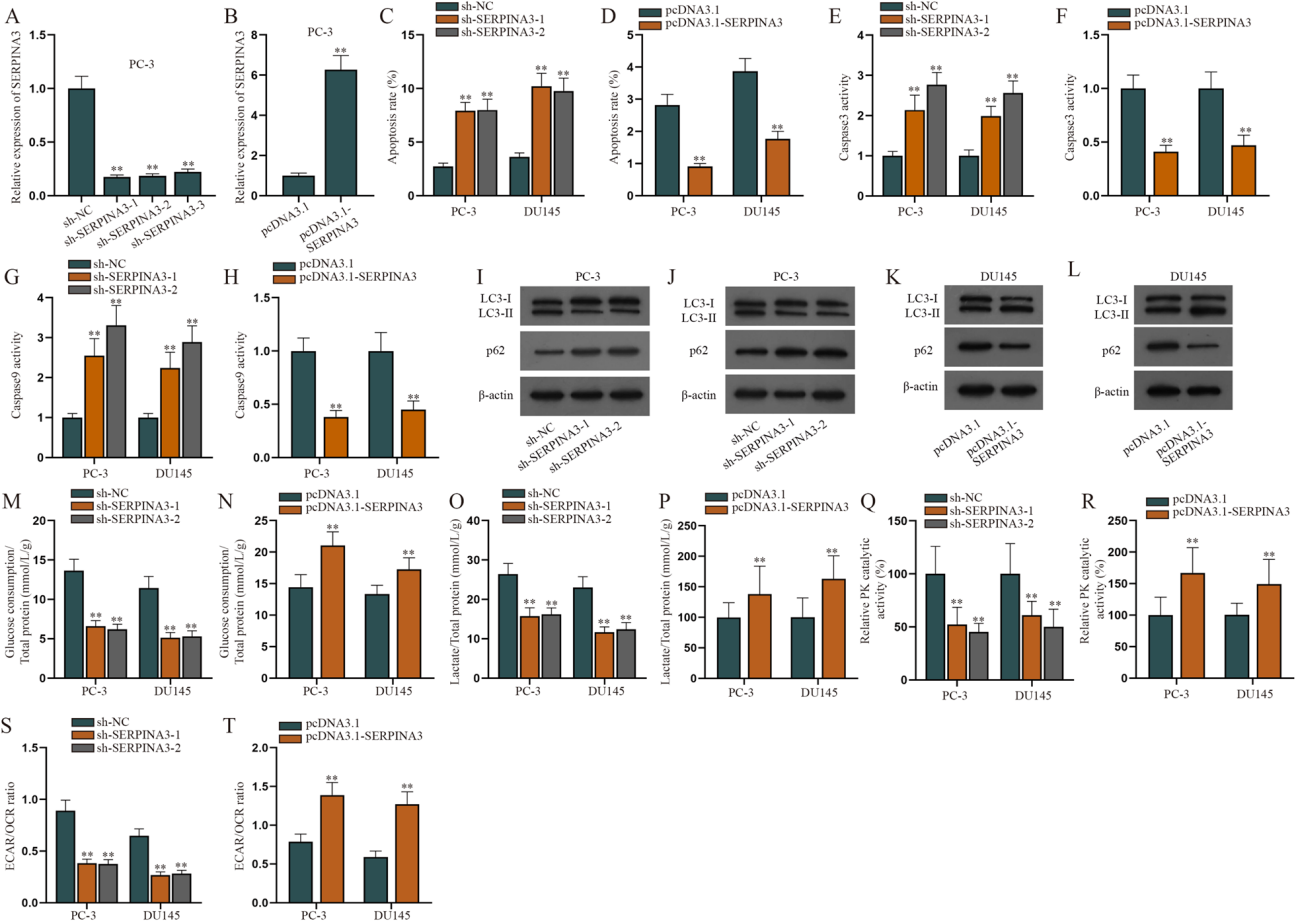
SERPINA3 inhibits cell apoptosis while promoting autophagy and aerobic glycolysis in PCa cells. **A**, **B** The knockdown efficiency of sh-SERPINA3-1/2/3 and overexpression efficiency of pcDNA3.1-SERPINA3 were examined through RT-qPCR. **C**, **D** An analysis of annexin V staining by flow cytometry was utilized to test apoptosis rate of PCa cells when SERPINA3 was down-regulated or overexpressed. **E**, **F** Caspase3 Activity Assay Kit was applied to examine Caspase3 activity when SERPINA3 was reduced or overexpressed in PCa cells. **G**, **H** Caspase9 activity was measured by means of Caspase9 Activity Assay Kit in PCa cells with SERPINA3 knockdown or elevation. **I**, **J** Western blot analysis was carried out to examine the expression of LC3-I, LC3-II and p62 when SERPINA3 was diminished in PCa cells. **K**, **L** The expression of LC3-I, LC3-II and p62 was analyzed by western blot assay when PCa cells were transfected with pcDNA3.1-SERPINA3. **M**, **N** The glucose consumption in PCa cells was tested when SERPINA3 was down-regulated or up-regulated. **O**, **P** Lactate content was quantified in PCa cells with SERPINA3 depletion or augment. **Q**, **R** PK activity was measured after PCa cells were co-cultured with sh-SERPINA3-1/2 or pcDNA3.1-SERPINA3. **S**, **T** ECAR/OCR value in PCa cells were examined upon SERPINA3 decline or overexpression. (*sh* short hairpin, *NC* negative control). ^**^P < 0.01

According to previous studies, SERPINA3 and pigment epithelium-derived factor (PEDF), members of serine protease inhibitor family, are involved in a variety of tumor progression [22, 23]. Since PEDF has been reported to exert function in regulating cell apoptosis, autophagy and aerobic glycolysis [24], we speculated that SERPINA3 may also play a vital role in PCa cells. To identify the regulatory pattern of SERPINA3, we examined the efficiency of sh-SERPINA3-1/2/3 and pcDNA3.1-SERPINA3 in PCa cells (Fig. 4A, B). Data from RT-qPCR indicated SERPINA3 was noticeably down-regulated after sh-SERPINA3-1/2/3 transfection and up-regulated after pcDNA3.1-SERPINA3 transfection. Specifically, sh-SERPINA3-1 and sh-SERPINA3-2 were involved in the following assays for being more efficient. An analysis of annexin V staining by flow cytometry was then utilized to test apoptosis rate of PCa cells when SERPINA3 was reduced or overexpressed (Fig. 4C, D). After that, Caspase3 and Caspase9 activity was detected when sh-SERPINA3 or pcDNA3.1-SERPINA3 was transfected into PCa cells (Fig. 4E–H), and results demonstrated that both Caspase3 and Caspase9 activities rose after SERPINA3 knockdown, whereas their activities declined after SERPINA3 up-regulation. As shown in Additional file 2: Figure S2B, C, the protein expression of Bcl-2 dropped, while that of Bax and Cleaved-PARP1 was lifted owing to SERPINA3 depletion. In addition, SERPINA3 augment brought about the opposite consequence of these apoptosis-related proteins (Additional file 2: Figure S2D, E). Based on the abovementioned findings, we drew the conclusion that SERPINA3 negatively regulated the apoptosis of PCa cells. Subsequently, western blot analysis was carried out to examine the expression of LC3-I, LC3-II and p62 when SERPINA3 was reduced or overexpressed in PCa cells (Fig. 4I–L). To reconfirm the role of SERPINA3 in PCa cell autophagy, rescue assays were conducted. As displayed in Additional file 2: Figure S2F, G, stimulated cell autophagy led by Rapamycin treatment was recovered due to SERPINA3 knockdown, which backed up the former results. Moreover, a series of rescue assays were implemented in PCa cells. It turned out down-regulating circSERPINA3 hampered cell autophagy while SERPINA3 augment reversed the former suppressive effect on cell autophagy (Additional file 2: Figure S2H, I). It was observed that when SERPINA3 was lessened, the expression of p62 and LC3-I increased while LC3-II decreased. On the contrary, SERPINA3 augment led to the opposite results, proving that SERPINA3 could positively regulate cell autophagy. Afterwards, we examined the glucose consumption in PCa cells and discovered that the glucose consumption decreased when SERPINA3 was down-regulated, but it increased when SERPINA3 was up-regulated (Fig. 4M, N). We also tested lactate content (Fig. 4O, P), PK activity (Fig. 4Q, R) and ECAR/OCR value (Fig. 4S, T) in PCa cells and found that SERPINA3 reduction led to the decrease in lactate content, PK activity and ECAR/OCR value of PCa cells. In contrast, SERPINA3 elevation had the reverse effect on aerobic glycolysis, which comprehensively certified that SERPINA3 positively regulated aerobic glycolysis in PCa cells. In conclusion, SERPINA3 inhibites cell apoptosis while promoting autophagy and aerobic glycolysis in PCa cells.

## Discussion

PCa is one of the most common malignancies occurring in the prostate gland [25]. Though improvements have been achieved in the diagnosis and therapy of PCa [26], more biomarkers of this disease remain to be identified. The role of circRNA has been investigated in many studies. For instance, up-regulated circZMIZ1 has been revealed to facilitate the proliferation of PCa cells and is a potential biomarker for PCa treatment [27]. The suppressive role of circRNA_100395 in the proliferation and metastasis of PCa cells has also been discovered recently [28]. In this study, circSERPINA3 was found to be overtly up-regulated in PCa cells compared to normal WPMY-1 cells by means of RT-qPCR. With the help of an analysis of annexin V staining by flow cytometry, western blot analysis and colorimetric assay kit, we found that circSERPINA3 could inhibit cell apoptosis while promoting autophagy and aerobic glycolysis (Warburg effect) in PCa cells. As reported, circSERPINA3 promotes cell proliferation and invasion in nasopharyngeal carcinoma [29]. Consistent with the former literature, our study also confirmed circSERPINA3 played a promoting role in PCa cells.

Recent evidence has highlighted the effect of ceRNA mechanism on PCa [30]. For example, circABCC4 functions as the ceRNA of miR-1182 to up-regulate FOXP4 expression, thus facilitating PCa progression [31]. Circ_KATNAL1 regulates PCa cell growth and invasion via targeting miR-145-3p/WISP1 pathway [32]. Herein, we also explored the potential ceRNA model of circSERPINA3 in PCa cells. We firstly identified circSERPINA3 was located both in the cytoplasm and nuclei of PCa cells using subcellular fractionation assay and FISH assay. Subsequently, miR-653-5p was determined to bind with circSERPINA3, and SERPINA3 was proved to be the target gene of miR-653-5p by means of bioinformatics tools and mechanism assays. After that, the results of rescue assays verified that circSERPINA3 could regulate SERPINA3 via competitively binding to miR-653-5p.

RBP plays a pivotal role in the modulation of multiple RNA processes, including splicing, translation stabilization and degradation of RNAs. Recent studies demonstrate that RBP emerges as major players in the development and spread of cancer [33]. For instance, the RBP FXR1 modulates PCa progression by promoting FBXO4 mRNA degradation [34]. Circ0005276 prompts the proliferation and migration of PCa cells by recruiting FUS to transcriptionally activate XIAP [35]. Moreover, the RBP SORBS2 restricts hepatocellular carcinoma tumourigenesis and metastasis through enhancing RORA mRNA stability [36]. In present study, we utilized starBase and mechanism assays and found that BUD13 could bind to both circSERPINA3 and SERPINA3. Further exploration demonstrated that down-regulated BUD13 could shorten the half-life of SERPINA3, which proved that BUD13 could enhance the mRNA stability of SERPINA3. Next, the results of a series of functional experiments showed SERPINA3 could inhibit cell apoptosis while promoting autophagy and aerobic glycolysis in PCa cells. The former research has proved SERPINA3 is up-regulated in colon cancer cells and boosts cell migratory and invasive abilities [23]. In line with this literature, our study also confirmed SERPINA3 served an oncogenic role in PCa cells. Finally, data from rescue assays indicated circSERPINA3 could regulate SERPINA3 to exert its function in PCa cell autophagy. Several studies have indicated some circRNAs have the ability of encoding proteins [37, 38], thus affecting cancer tumorigenesis and progression. We planned to investigate whether circSERPINA3 could also encode proteins to initiate PCa onset in the future.

## Conclusion

To sum up, our study first proved that circSERPINA3 could regulate SERPINA3-mediated apoptosis, autophagy and aerobic glycolysis of PCa cells by competitively binding to miR-653-5p and recruiting BUD13. In spite of the lack of in vivo assay and clinical samples, we still hope our study could provide a novel insight for PCa diagnosis and treatment.

## Supplementary Information

**Additional file 1: Figure S1.** (A) The expression of circSERPINA3 was detected via RT-qPCR after WPMY-1 cells were transfected with pcDNA3.1-circSERPINA3. (B) WPMY-1 cell apoptosis was detected with the help of an analysis of annexin V staining by flow cytometry after circSERPINA3 overexpression. (C) Caspase3 Activity Assay Kit was applied to examine Caspase3 activity when circSERPINA3 was up-regulated in WPMY-1 cells. (D) After circSERPINA3 elevation, Caspase9 activity in WPMY-1 cells was detected by Caspase9 Activity Assay Kit. (E–F) The expression of apoptosis-associated proteins (Bcl-2, Bax and Cleaved-PARP1) was analyzed by western blot in PCa cells with sh-circSERPINA3-1/2 transfection. (G-H) Western blot was implemented to examine the expression of apoptosis-associated proteins in PCa cells transfected with pcDNA3.1-circSERPINA3. (I) The levels of cell autophagy-related proteins (LC3-I, LC3-II and p62) was detected via western blot in WPMY-1 cells with circSERPINA3 overexpression. (J-K) Cell autophagy was evaluated by means of western blot in PCa cells under different conditions (Rapamycin treatment or circSERPINA3 reduction). (L) Glucose consumption in WPMY-1 cells was tested after circSERPINA3 overexpression. (M) Lactate content was detected in WPMY-1 cells transfected with pcDNA3.1-circSERPINA3. (N) PK activity was examined in WPMY-1 cells with circSERPINA3 up-regulation. (O) ECAR/OCR value in WPMY-1 cells was tested under the condition of circSERPINA3 elevation. (sh: short hairpin; NC: negative control). ^**^P < 0.01.

**Additional file 2: Figure S2.** (A) Candidate RBP likely binding to circSERPINA3 (conditions: CLIP-Data >  = 2 and ClusterNum >  = 15) and potential RBP likely combining with SERPINA3 (CLIP-Data >  = 2 and ClusterNum >  = 15) were predicted from starBase. (B-C) The protein levels of Bcl-2, Bax and Cleaved-PARP1 were detected by western blot in PCa cells transfected with sh-SERPINA3-1/2. (D-E) The expression of apoptosis-related proteins was examined after SERPINA3 augment in PCa cells by means of western blot. (F-G) Cell autophagy was evaluated via western blot in PCa cells under different conditions (Rapamycin treatment or SERPINA3 reduction). (H-I) Western blot was utilized to analyze the expression of autophagy-related proteins in PCa cells with different transfection (sh-NC, sh-circSERPINA3-1, sh-circSERPINA3-1 + pcDNA3.1 and sh-circSERPINA3-1 + pcDNA3.1-SERPINA3). (sh: short hairpin; NC: negative control).

## Data Availability

Not applicable.

## Abbreviations

circRNA: Circular RNA
mRNA: Messenger RNA
miRNA: MicroRNA
PCa: Prostate cancer
ceRNAs: Competing endogenous RNAs
ATCC: American Type Culture Collection
DMEM: Dulbecco’s Modified Essential Medium
RT-qPCR: Quantitative real-time RT-PCR
FBS: Fetal bovine serum
RIN: RNA integrity number
cDNA: Complementary DNA
WT: Wild-type
Mut: Mutant
SDS-PAGE: Sodium dodecyl sulfate polyacrylamide gel electrophoresis
PVDF: Polyvinylidene fluoride
RIP: RNA immunoprecipitation assay
RBP: RNA binding protein
FISH: Fluorescence in situ hybridization
SD: Standard deviation
AGE: Agarose gel electrophoresis
PK: Pyruvate kinase
ECAR/OCR: Extracellular acidification rate/oxygen consumption rate
PEDF: Pigment epithelium-derived factor
ECL: Enhanced chemiluminescence
