# Supplementary figures and images for: CircSERPINA3 regulates SERPINA3-mediated apoptosis, autophagy and aerobic glycolysis of prostate cancer cells by competitively binding to MiR-653-5p and recruiting BUD13
Source: J Transl Med. 2021 Dec 3;19:492. doi: 10.1186/s12967-021-03063-2 (PMC8642898; doi:10.1186/s12967-021-03063-2)

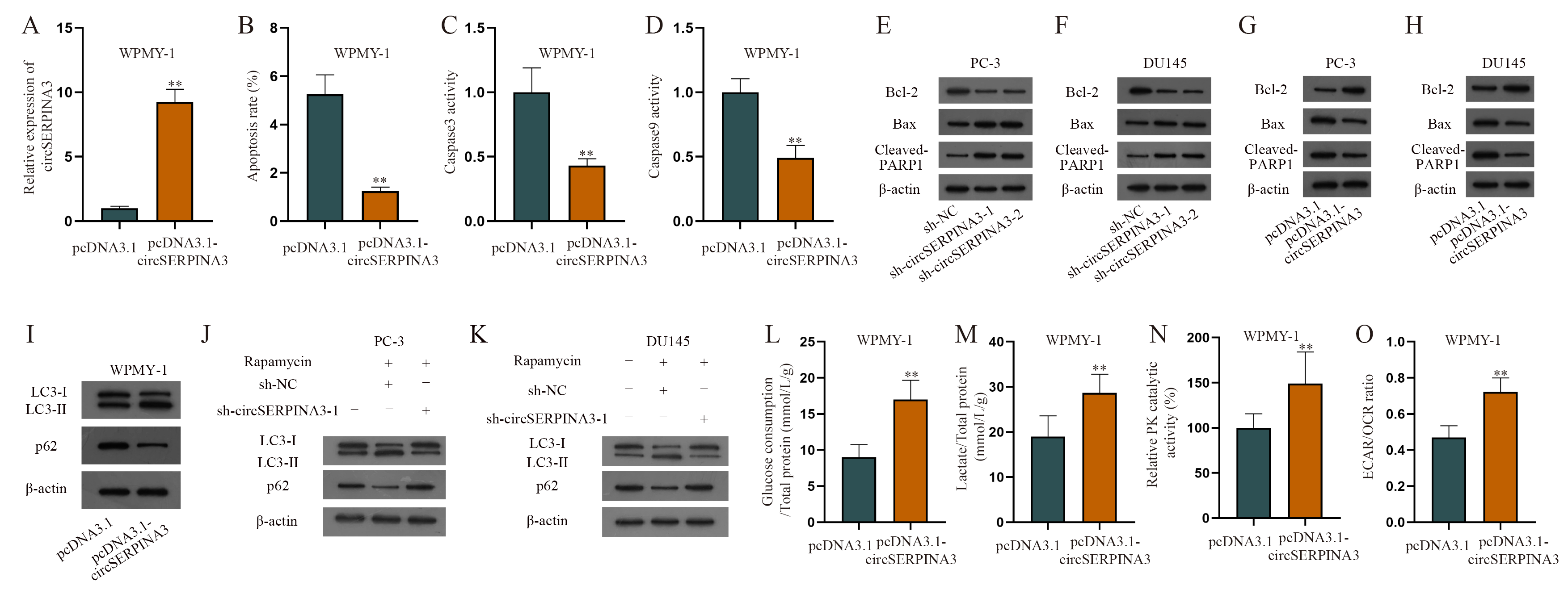

Supplement: Supplementary file 1 — Additional file 1: Figure S1. (A) The expression of circSERPINA3 was detected via RT-qPCR after WPMY-1 cells were transfected with pcDNA3.1-circSERPINA3. (B) WPMY-1 cell apoptosis was detected with the help of an analysis of annexin V staining by flow cytometry after circSERPINA3 overexpression. (C) Caspase3 Activity Assay Kit was applied to examine Caspase3 activity when circSERPINA3 was up-regulated in WPMY-1 cells. (D) After circSERPINA3 elevation, Caspase9 activity in WPMY-1 cells was detected by Caspase9 Activity Assay Kit. (E–F) The expression of apoptosis-associated proteins (Bcl-2, Bax and Cleaved-PARP1) was analyzed by western blot in PCa cells with sh-circSERPINA3-1/2 transfection. (G-H) Western blot was implemented to examine the expression of apoptosis-associated proteins in PCa cells transfected with pcDNA3.1-circSERPINA3. (I) The levels of cell autophagy-related proteins (LC3-I, LC3-II and p62) was detected via western blot in WPMY-1 cells with circSERPINA3 overexpression. (J-K) Cell autophagy was evaluated by means of western blot in PCa cells under different conditions (Rapamycin treatment or circSERPINA3 reduction). (L) Glucose consumption in WPMY-1 cells was tested after circSERPINA3 overexpression. (M) Lactate content was detected in WPMY-1 cells transfected with pcDNA3.1-circSERPINA3. (N) PK activity was examined in WPMY-1 cells with circSERPINA3 up-regulation. (O) ECAR/OCR value in WPMY-1 cells was tested under the condition of circSERPINA3 elevation. (sh: short hairpin; NC: negative control). **P < 0.01. [file 12967_2021_3063_MOESM1_ESM.tif]

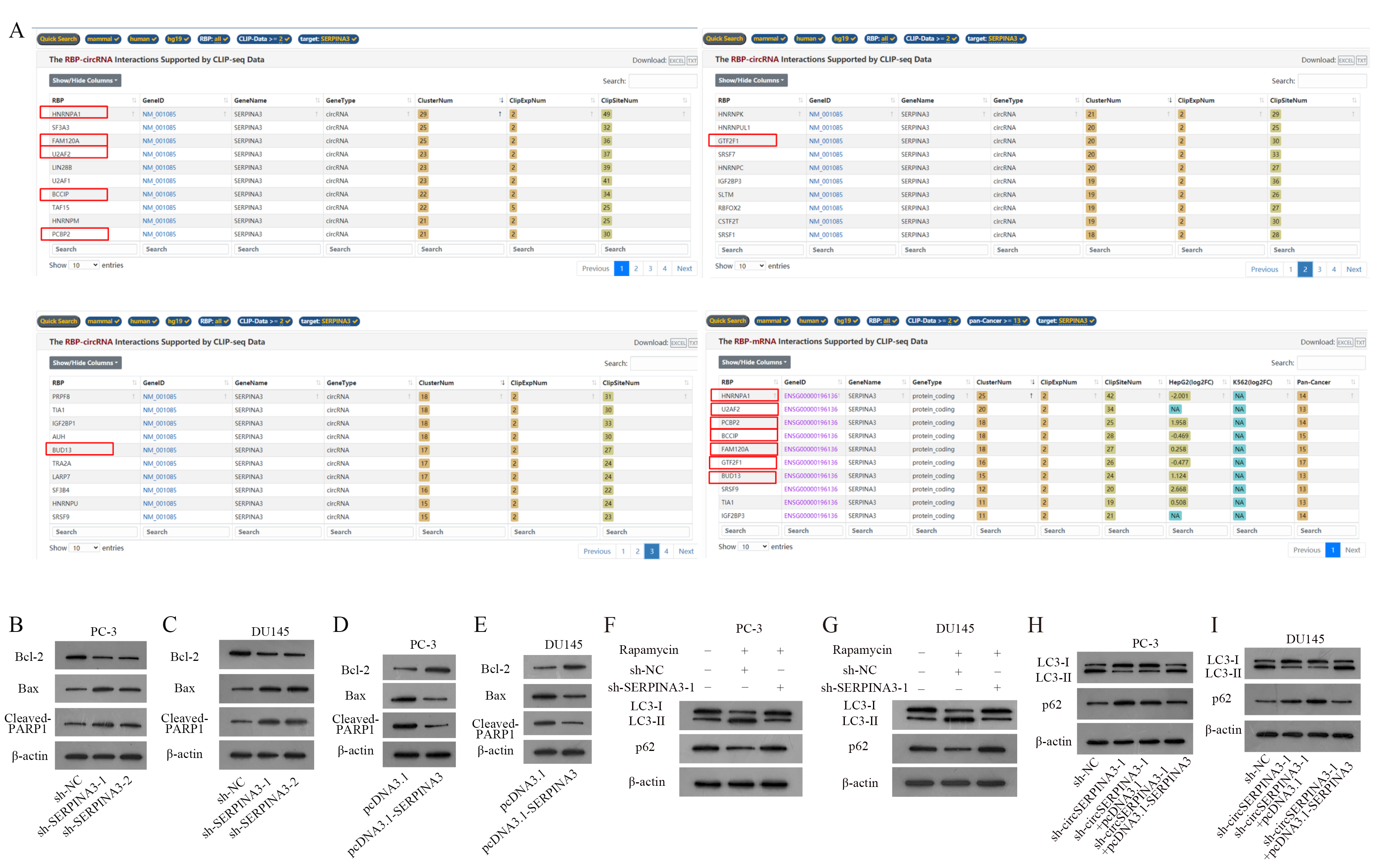

Supplement: Supplementary file 2 — Additional file 2: Figure S2. (A) Candidate RBP likely binding to circSERPINA3 (conditions: CLIP-Data > = 2 and ClusterNum > = 15) and potential RBP likely combining with SERPINA3 (CLIP-Data > = 2 and ClusterNum > = 15) were predicted from starBase. (B-C) The protein levels of Bcl-2, Bax and Cleaved-PARP1 were detected by western blot in PCa cells transfected with sh-SERPINA3-1/2. (D-E) The expression of apoptosis-related proteins was examined after SERPINA3 augment in PCa cells by means of western blot. (F-G) Cell autophagy was evaluated via western blot in PCa cells under different conditions (Rapamycin treatment or SERPINA3 reduction). (H-I) Western blot was utilized to analyze the expression of autophagy-related proteins in PCa cells with different transfection (sh-NC, sh-circSERPINA3-1, sh-circSERPINA3-1 + pcDNA3.1 and sh-circSERPINA3-1 + pcDNA3.1-SERPINA3). (sh: short hairpin; NC: negative control). [file 12967_2021_3063_MOESM2_ESM.tif]
